# Spinale Ozontherapie

**DOI:** 10.1007/s00117-021-00878-4

**Published:** 2021-07-09

**Authors:** F. Ahlhelm, R. Rotzinger, M. Heesen, H. Gebhard, R. Omidi

**Affiliations:** 1 Abteilung Neuroradiologie, Zentrum für Bildgebung, Kantonsspital Baden AG, Baden, Schweiz; 2 Abteilung Anästhesie und Intensivmedizin, Kantonsspital Baden AG, Baden, Schweiz; 3 Klinik für Traumatologie, Universitätsspital Zürich ZH, Zürich, Schweiz; 4 Abteilung Wirbelsäulenchirurgie, Kantonsspital Baselland BL, Baselland, Schweiz

**Keywords:** Rückenschmerz, Schmerztherapie, Wirbelsäulenchirurgie, Interventionelle Neuroradiologie, Ozon, Back pain, Pain therapy, Spinal surgery, Interventional neuroradiology, Ozone

## Abstract

**Klinisches/methodisches Problem:**

Technische Fortschritte auf dem Gebiet der spinalen interventionellen Neuroradiologie ermöglichen es, eine breite Palette an gezielten, minimal-invasiven Behandlungsoptionen einschließlich der spinalen Ozontherapie beim Rückenschmerz anzuwenden. Dieser Beitrag gibt eine Übersicht der biochemischen, molekularen, immunologischen und pharmazeutischen Mechanismen sowie Applikationstechniken der gezielten Ozontherapie.

**Radiologische Standardverfahren:**

Zum Einsatz kommen die Computertomographie (CT) sowie konventionelle Röntgenaufnahmen (Durchleuchtung).

**Leistungsfähigkeit:**

Die CT-gesteuerten Interventionen (epidural, periradikuläre, Facettengelenk und intradiskal) haben den höchsten Stellenwert und haben sich historisch durchgesetzt. Durchleuchtungsgesteuerte Verfahren können ebenfalls eingesetzt werden.

**Schlussfolgerung:**

Die Ozontherapie liefert vielversprechende Ergebnisse. Der Beitrag soll dazu dienen, Informationen über die Grundlagen dieser Technik(en) zu vermitteln.

## Abstract (en)

**Clinical/methodological problem:**

Technical advances in the field of spinal interventional neuroradiology allow the application of a wide range of targeted, minimally invasive treatment options including targeted spinal ozone therapy for back pain. This article provides an overview of the biochemical, molecular, immunologic, and pharmaceutical mechanisms and delivery techniques of targeted ozone therapy.

**Standard radiological methods:**

Computed tomography (CT), conventional X‑ray (fluoroscopy).

**Performance:**

Computed tomography-guided interventions (epidural, periradicular, facet joint, and intradiscal) have the highest place value and have historically prevailed. Fluoroscopy-guided procedures may also be used.

**Practical recommendations:**

Ozone therapy provides promising results. The article is intended to provide information on the basics of the technique(s).

## Hintergrund

Der *Rückenschmerz* ist eine verbreitete Volkskrankheit. Frauen sind etwas häufiger betroffen als Männer [1]. Die Prävalenz beträgt etwa 33 %, auf Lebenszeit 84 % [2]. In Deutschland leiden etwa 20 % der Erwachsenen an intensiven oder einschränkenden Rückenschmerzen, 10 % geben Schmerzen hoher Intensität unter teils invalidisierenden Beeinträchtigungen an [3, 4]. Vor diesem Hintergrund sind hohe volkswirtschaftliche Kosten mit dem Phänomen *Rückenschmerz* verbunden. Die direkten Kosten werden in der Bundesrepublik auf jährlich bis zu 7000 € pro Patienten beziffert, wobei die indirekten Kosten durch folgebedingte Arbeitsausfälle und Erwerbsunfähigkeit mit bis zu 75 % noch deutlich höher anzusetzen sind [5].

Die genaue Ursache von Rückenschmerzen ist oft schwer zu lokalisieren. Daher stellen sie ein Problem für Diagnose und Behandlung dar [6].

Neben unspezifischen Rückenschmerzen, die nicht selten auf körperliche Fehlbelastungen oder Bewegungsmangel zurückzuführen sind, gibt es eine Reihe spezifischer Rückenschmerzen [6]. Hierunter fallen u. a. Schmerzen durch Bandscheibenvorfälle, Wirbelgleiten, Facettengelenkarthrosen oder Spinalkanalstenosen sowie myofasziale Ursachen. Meist treten diese Veränderungen im Rahmen von Alterungsprozessen auf, sie können aber auch direkte oder indirekte Folge eines vorausgegangenen Traumas oder nach Wirbelsäuleneingriffen (im Sinne eines Gewebetraumas einschließlich Kollateralschäden durch den Operationszugang), z. B. beim „failed back surgery syndrome“ (FBSS), sein [7, 8].

## Pathologie des (Rücken‑)Schmerzes

Die Ursprünge von Rückenschmerzen sind divers und können auf verschiedene Gewebe, wie Muskeln, Bänder, Gelenke oder Gefäße, zurückgehen [9]. Zu unterscheiden sind nozizeptive (entzündlicher Schmerz, Frakturschmerz, Arthritis etc.) von neuropathischen Schmerzen (posttherapeutische Neuropathie, diabetische Polyneuropathie, posttraumatische Nervenschädigung, Phantomschmerz) und deren Mischformen (lumbo- oder zervikoradikuläre Schmerzen, posttraumatischer Schmerz; [10, 11]). Der Vollständigkeit halber sei auch der noziplastische Schmerz erwähnt, den die *International Association for the Study of Pain* (IASP) 2017 als weitere Schmerzkategorie eingeführt hat.

Der nozizeptive Schmerz geht auf die sog. *Urnoxen* (wie z. B. Säure, spitzes und stumpfes Trauma, Hitze oder Kälte) zurück und dient dem Körper allgemein als wichtiger Warnhinweis für eine mögliche Schädigung der beteiligten Strukturen. Zwischen Einwirken und Gewebeschädigung läuft dabei eine komplexe biochemische und elektrophysiologische Kaskade ab, die in 4 Prozesse unterteilt werden kann [10, 11]:

1. *Transduktion:* Aktivierung der Nozizeptoren durch sog. Urnoxen einschließlich mechanischer, chemischer und thermischer Stimuli und Übertragung an die afferenten Nerven durch Änderung des Membranpotenzials.
2. *Transmission:* Weiterleitung des afferenten nozizeptiven Impulses im peripheren und zentralen Nervensystem unter Beteiligung folgender Neuronen:
  1. Neuron: peripherer Nerv – Spinalganglion – Hinterhorn,
  2. Neuron: Hinterhorn (Substantia gelatinosa) – Rückenmark – Thalamus,
  3. Neuron: Thalamus – Kortex,
  4. Neuron: Kortex.
3. *Modulation: *Bearbeitung der Informationen durch spezifische neuronale Hemmsysteme.
4. *Perzeption/Interozeption:* veränderte Aktivität im zentralen Nervensystem (ZNS). Bewusste oder unbewusste subjektive Schmerzwahrnehmung und Schmerzäußerung, einschließlich willkürlicher und autonomer Reaktionen.

Der neuropathische Schmerz hat keine Warnfunktion. Er ist durch die bereits eingetretene Schädigung des somatoafferenten Systems gekennzeichnet. Es handelt sich um eine Dysfunktion des Nervensystems an sich. Der neuropathische Schmerz wird als anfallsartig einschießend, eher brennend, kribbelnd und dumpf beschrieben [11].

Als noziplastische Schmerzen werden Schmerzen bezeichnet, die durch eine veränderte Nozizeption bedingt sind, jedoch ohne Nachweis einer bestehenden oder potenziellen Gewebeschädigung als Ursache einer Aktivierung peripherer Nozizeptoren und ohne Hinweise auf eine Erkrankung oder Schädigung des somatosensorischen Systems als Ursache der Schmerzen [12].

Bei chronischen Rückenschmerzen, insbesondere bei Radikulopathien, handelt es sich meist um Mischformen des nozizeptiven und neuropathischen Schmerzes [10].

## Invasive Behandlung des Rückenschmerzes

Die Behandlungsoptionen des Rückenschmerzes richten sich nach deren Ursache. Das therapeutische Vorgehen ist von den subjektiven Beschwerden sowie vor allem von der neurologischen Symptomatik, nicht dem Ausmaß der radiologischen Befunde, abhängig. Die Behandlungsoptionen umfassen neben der medikamentösen und konservativen Therapie verschiedene invasive (offen-chirurgische und minimal-invasive) Verfahren [13, 14].

Wenn immer möglich, sollten die bildgesteuerten minimal-invasiven Verfahren in einem multimodalen Therapiekonzept Anwendung finden. Im Fall sog. „red flags“ ist eine notfallmäßige Abklärung einschließlich operativer Versorgung notwendig. Es handelt sich um anamnestische und/oder klinische Warnhinweise, die auf einen gefährlichen Krankheitsverlauf bei Rückenschmerzen hindeuten können (z. B. das Konus-Kauda-Syndrom im Rahmen einer Diskusherniation oder Fieber im Rahmen einer Spondylodiszitis; [11, 15]).

Aufgrund des geringeren Gewebetraumas sind, sofern möglich, minimal-invasive Verfahren den offen-chirurgischen Operationsverfahren vorzuziehen. Für die Behandlung von Bandscheibenvorfällen hat sich die mikrochirurgische Diskektomie als Standard gegenüber der offen-chirurgischen Operation etabliert. Aus radiologischer Sicht ist jedoch zu betonen, dass auch der mikrochirurgische Zugang mit Fibrosierungen der myofaszialen Strukturen, Denervationsverletzungen der Muskulatur sowie Störungen des osteodiskoligamentären Gefüges im Rahmen einhergeht (s. oben FBSS) und eine größere Gewebeschädigung als der alternative minimal-invasive Zugang mit einer 22G-Nadel bedeutet [11].

### Minimal-invasive Verfahren

Bei den minimal-invasiven Techniken stehen diagnostische Infiltrationen (mittels Applikation von Lokalanästhetika) und therapeutische Infiltrationen (in Deutschland und der Schweiz vor allem Kortisonpräparate) im Vordergrund [16].

Bei pseudoradikulären Schmerzen, dem sog. *Facettensyndrom* (z. B. im Rahmen einer Entzündung der Zygapophysealgelenke) hat sich die gezielte intra- bzw. periartikuläre Infiltration der Gelenke mit Kortikoiden/Lokalanästhetika bewährt. Als symptomatische und weniger kausale Alternative stehen, bei positiver diagnostischer/therapeutischer Facettengelenkblockade und persistierenden Beschwerden, zudem die Kryodenervation und Thermokoagulation des Ramus dorsalis medialis des Spinalnerven zur Verfügung [16].

Die etablierten minimal-invasiven Verfahren zur Behandlung des Epiduralraums und der Nervenwurzeln umfassen die periradikuläre bzw. transforaminale Infiltration, sowie die interlaminäre Infiltration und die Sakralblockade [16].

Erweiterte minimal-invasive Verfahren schließen zudem die Behandlung der Bandscheibe und des Wirbelknochens mit ein. Hierzu zählt insbesondere die intradiskale Kortisoninjektion, die Kryoablation bzw. die Chemonukleolyse (z. B. mittels Chymopapain; [17, 18]).

Als wichtige Option im Rahmen minimal-invasiver Therapien wird weltweit zudem die spinale Ozontherapie eingesetzt. Sie ist im europäischen Raum vor allem in Italien etabliert [17] und kann z. B. analog zur chemischen Nukleolyse intradiskal Anwendung finden [18].

Im Folgenden soll im Detail näher auf die verschiedenen therapeutischen Ansätze der minimal-invasiven Ozontherapie eingegangen werden.

## Ozontherapie

### Pharmakokinetik und biologische Prozesse

Ozon (O_3_) ist ein aus 3 Sauerstoffatomen aufgebautes Gas. Es kommt in größeren Mengen natürlich in höheren Schichten der Erdatmosphäre vor. In der Stratosphäre besitzt es eine Konzentration von etwa 16–20 mg/m^3^. In Wasser (H_2_O) besitzt Ozon gegenüber molekularem Sauerstoff (O_2_) eine etwa 10-fach höhere Löslichkeit (etwa 50 ml O_3_/100 ml H_2_O bei 0 °C), d. h. es reagiert sofort bei Kontakt mit *wasserreichen* Geweben. Ozon ist nach Fluor und Persulfat eines der stärksten bekannten Oxidationsmittel. Unter normalen Bedingungen ist Ozon nicht lagerungsfähig (Halbwertszeit etwa 1 h bei Raumtemperatur) und muss vor Ort elektrophoretisch mittels eines Ozongenerators mittels Hochvolt-Technik (5–13 Megavolt) hergestellt werden. Dabei wird ein Gasgemisch mit etwa 95 % Sauerstoffanteil und etwa 5 % Ozon gewonnen, wobei darauf zu achten ist, dass das Gasgemisch nicht inhaliert wird [19].

Da Ozon in Wasser gelöst ausgesprochen reaktionsfreudig ist, erzeugt es in biologischen Systemen als sog. „Pro-Drug“ einen hohen oxidativen Stress, insbesondere in Systemen, die selbst einen hohen Wasseranteil besitzen. Der Wirkmechanismus des Ozons im biologischen Gewebe ist aufgrund der Vielzahl der verschiedenen beteiligten Systeme komplex. Zusammengefasst kommt es zu einer Hochregulation des endogenen antioxidativen Systems, zu einer Aktivierung des Immunsystems und zu einer Unterdrückung verschiedener Entzündungs- und Schmerzmediatoren (vermittelt durch *Hypoxia inducible factor-1α *(HIF1A), *Nuclear factor of activated T‑cells* (NFAT), *Nuclear factor-erythroid 2‑related factor 2* (NRF2) bzw. *Antioxidant response element* (ARE) *activated Protein‑1; *[19, 20]).

**Figure 1:**
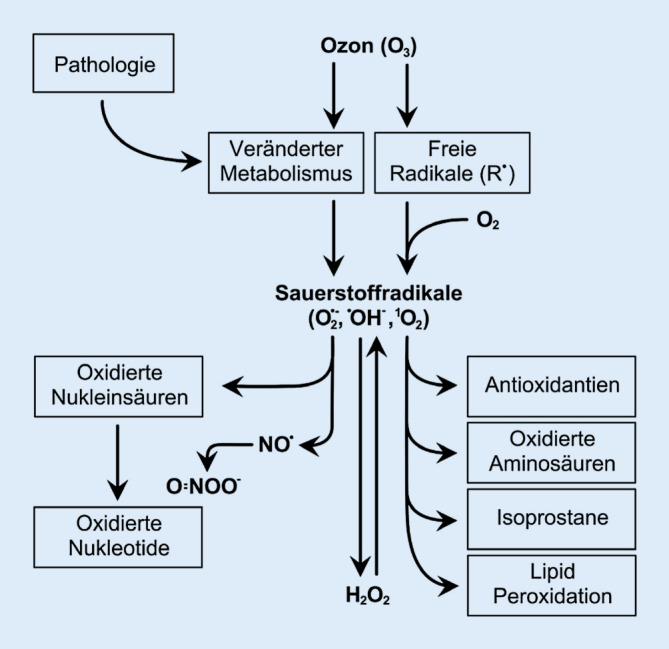

In Abb. 1 wird eine Übersicht über die Interaktion von Ozon in biologischen Systemen gegeben [18].

Man geht außerdem davon aus, dass es indirekt über die Bildung von Sauerstoffradikalen wie Wasserstoffperoxid (H_2_O_2_) und Lipoperoxiden mit deren Oxidationsprodukten zur Aktivierung des deszendierenden antinozizeptiven Systems kommt, mit analgesierender Wirkung [21]. Auf Ebene des 2. und 3. Neurons (z. B. Thalamus und Kortex, s. oben) blockieren Endorphine zentral die Transmission. Zudem vermutet man über die Oxygenierung positive indirekte Effekte peripher auf die Muskelrelaxation und Gefäßdilatation [19, 21].

### Durchführung und therapeutische Aspekte

Abgesehen von den bekannten Risiken, die minimal-invasiven Eingriffen an der Wirbelsäule inhärent sind, ist die korrekt durchgeführte, intradiskale Ozontherapie unter strenger Berücksichtigung geeigneter Ozonkonzentrationen risikoarm und gut verträglich [21]. Bis heute sind ernste Komplikationen selten beschrieben. Schwächeanfälle bzw. vasovagale Reaktionen können selten auftreten [18]. Es empfiehlt sich neben einer langsamen Injektion und Beachtung von Höchstkonzentrationen ein entsprechend angepasstes Blutdruck‑, Frequenz-Monitoring mittels EKG und Blutsättigungskontrolle sowie ein intravenöser Zugang, auch zur Schmerzmedikation und ggf. Anxiolyse bzw. Sedierung.

Allergische Reaktion auf eine Ozonanwendung sind nicht zu erwarten, da sowohl Ozon als auch Sauerstoff keine allergene Potenz besitzen. Die fehlende allergische Potenz ist neben den geringen Kosten des Ozons (abgesehen von der Gerätebeschaffung) ein weiterer Vorteil im Vergleich zu den Medikamenten wie Lokalanästhetika und sogar Glukokortikoiden.

**Figure 2:**
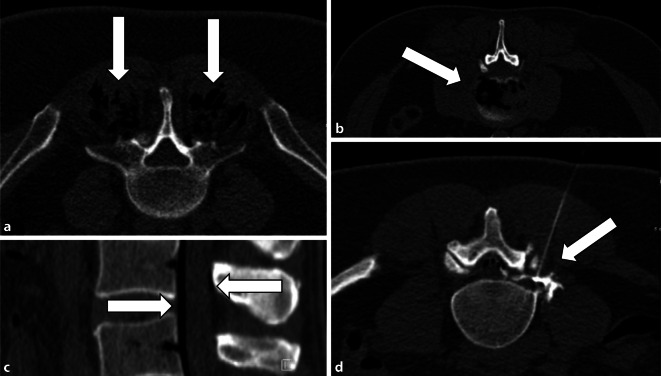

Spinale Ozontherapien können nach Schmerzursache unterteilt werden (Abb. 2):

- intramuskulär,
- intraartikulär,
- intradiskal (Ozonnukleolyse),
- epidural (interlaminär oder transforaminal).

### Intramuskuläre Ozontherapie

Der einfachste Ansatz der Ozontherapie geht zurück auf den Italiener und Vorreiter der Ozontherapie C. Verga. Bereits 1988 führte er die intramuskuläre Ozontherapie zur einfachen, risikoarmen und kostengünstigen Behandlung bei Rückenschmerz ein [22].

Es werden 5–10 ml einer 20 %igen Sauerstoff-Ozon-Lösung direkt in Trigger-Punkte der Paravertebralmuskeln injiziert. Eine Bildsteuerung des ambulanten Eingriffs ist dazu nicht erforderlich. Nach der Behandlung wird der Patient etwa 30 min überwacht [22].

### Intraartikuläre Ozontherapie

Bei der intraartikulären Ozontherapie stehen klinisch Lumbalgie und pseudoradikuläre Schmerzen im Vordergrund, die bei langem Stehen und Gehen, insbesondere unter Reklination und Rotation in Richtung des pathologischen Gelenks, exazerbieren. Häufig sind die Beschwerden mit einer Hyperalgesie sowie lokaler Druckschmerzhaftigkeit vergesellschaftet [23].

Der Eingriff kann mittels Fluoroskopie oder CT durchgeführt werden. In der Durchführung und therapeutischen Effektivität entspricht die intraartikuläre Ozontherapie weitgehend der Standardtechnik mittels intra- bzw. periartikulärer Injektionsbehandlung mit Steroiden und Lokalanästhetika. Wie bei der epiduralen und intradiskalen Behandlung kann aufgrund des Negativkontrastes durch das O_3_/O_2_-Gasgemsich auf eine intravenöse Röntgenkontrastmittelgabe verzichtet werden [23].

### Intradiskale Ozontherapie

**Figure 3:**
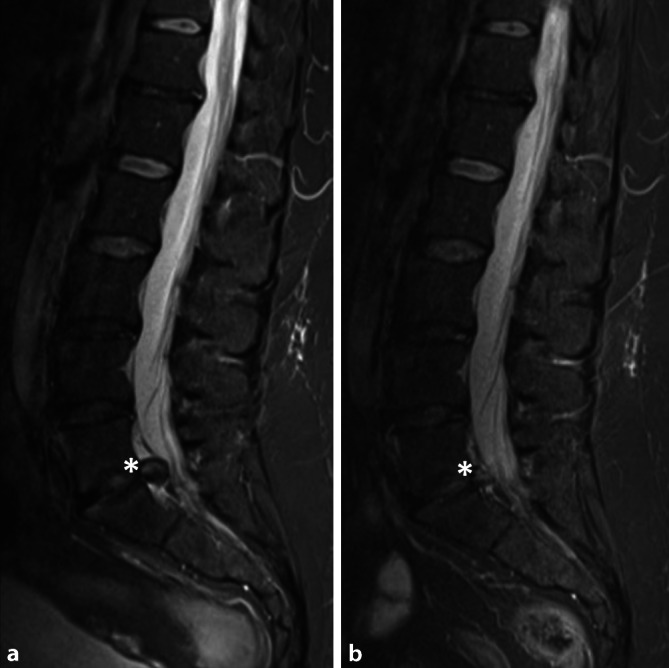

Die intradiskale Ozontherapie kann eine Behandlungsalternative zur mikrochirurgischen Diskektomie bzw. zur minimal-invasiven Chemonukleolyse (z. B. mittels Chymopapain) darstellen. Ozon fördert in der Bandscheibe über H_2_O_2_ und OH^-^den Abbau verschiedener Kollagene des Nucleus pulposus (Kollagen Typ I und Typ II) und die Matrixdegeneration. So führt es zu einer beschleunigten Resorption und Volumenminderung des geschädigten Bandscheibengewebes und zu einer verminderten mechanischen Reizung und letztlich fokalen Entzündung des umliegenden Gewebes (Abb. 3). Zudem vermutet man über die Freisetzung bestimmter Gewebefaktoren (über *Transforming growth factor-beta 1* [TGF-beta 1] bzw. *Basic fibroblast growth factor* [bFGF]) einen positiven Effekt auf die Reorganisation und beginnende Fibrosierung des Bandscheibengewebes [19].

Die geschädigte Bandscheibe wird über den posterolateralen Kambin-Zugang unter bildgebender Kontrolle mittels Durchleuchtung oder CT mit der 22G-Nadel punktiert. Anschließend werden 3–10 ml Sauerstoff-Ozon-Lösung langsam unter Bildkontrolle injiziert. Generell reichen im Bandscheibengewebe bereits niedrige O_3_-Konzentrationen, um ausreichend oxidativen Stress für eine adäquate Reaktion zu induzieren (etwa 20 μg/ml; [24]). Einige Autoren empfehlen eine limitierte Konzentration von 18–25 μg/ml, da Konzentrationen > 20 μg/ml als schmerzhaft erlebt werden können und die Gefahr vasovagaler Reaktionen steigt. Konzentrationen über 30 μg/ml sollten nicht überschritten werden. Bei Dosierungen < 18 μg/ml sinkt die Effektivität der Behandlung. Der Patient wird über etwa 3 h nach dem Eingriff überwacht [18]. Die Behandlung kann entsprechend im ambulanten Rahmen durchgeführt werden [25].

### Epidurale Ozontherapie

Über die gezielte epidurale Ozonbehandlung beim FBSS gibt es noch nicht viele publizierte Studien. Analog zu den oben beschriebenen prinzipiellen Mechanismen reagiert das Ozon mit organischen Molekülen und hat über mehrere enzymatische und nichtenzymatische Puffersysteme eine entsprechende entzündungshemmende und schmerzstillende Wirkung. Aktuelle Hypothesen legen nahe, dass eine chemische Adhäsiolyse der assoziierten Narbenfibrose und die Dehydrierung des Bandscheibengewebes verstärkt wird [26].

Bei der epiduralen Behandlung unterscheidet man zwischen dem interlaminären und transforaminalen Zugang (s. oben). Der epidurale Zugang über den Hiatus sacralis scheint für die Ozontherapie aktuell nicht relevant. Bei Einsatz von Kortikoiden und Lokalanästhetika scheint der transforaminale Zugang in der Regel dem interlaminären Zugang überlegen. Bei Einsatz von Ozon ist hingegen unklar, welcher Zugang zu bevorzugen ist. Wahrscheinlich spielt hier die Lokalisation der Fibrosierung eine Rolle (z. B. einseitig umschrieben mit Bezug zum Foramen vs. zirkumferentes Narbengewebe).

Die Ozontherapie kann auch mit Steroiden und Lokalanästhesie kombiniert werden. Sie scheint bei der intradiskalen und transforaminalen Behandlung der reinen Behandlung mit Steroid und Lokalanästhetikum überlegen [27]. So berichtete beispielsweise die Arbeitsgruppe um Bonetti et al., dass eine mit Ozon behandelte Gruppe innerhalb der 6‑monatigen Nachbeobachtungszeit eine deutlichere Verbesserung zeigte als Patienten, die nur eine Steroidinjektion erhielten. Zusätzlich zu der Rolle von Ozon bei FBSS berichtet die Arbeitsgruppe auch eine analgetische Wirkung bei Lumbago mit Radikulopathie [28].

## Fazit für die Praxis

- Die Ozontherapie liefert vielversprechende Ergebnisse, ihr wird jedoch in Fachkreisen immer noch mit Vorbehalten begegnet.
- Der Beitrag soll dazu dienen, diese Vorbehalte zu relativieren und Informationen über Grundlagen dieser Technik(en) vermitteln.
